# Connections Network: Harnessing the Collective Influence of Grassroots Leaders to Address Health-Related Problems in Hawkins and Hancock County, TN

**DOI:** 10.13023/jah.0403.08

**Published:** 2023-01-01

**Authors:** Jodi L. Southerland, Teresa Buttry, Connie Johnson, Sheldon Livesay, Lisa Nichols, Priscilla Rogers

**Affiliations:** Of One Accord Inc, southerlanjl@etsu.edu; United Way of Hawkins County; Hope Helps TN; Of One Accord Inc

**Keywords:** Appalachia, collaboration, nonprofits, rural health

## Abstract

In March 2021, grassroots leaders in two counties in northeast Tennessee formed a new network called *Connections*. Leaders are working to strengthen the capacity of the network and member organizations by promoting partnerships as vital to address effectively rural social determinants of health. Connections provides network members with capacity-building tools and resources, including two funding opportunities, to achieve their missions and sustain impact. Network members are also aligning around common goals to address the socioeconomic conditions affecting health outcomes. Connections will utilize findings from network activities and collaborations to identify synergies that can accelerate improvements in community health and well-being.

Rural and low-resource communities rely on the nonprofit sector to deliver vital services that address the social determinants of health. Rural nonprofits face myriad challenges that may negatively impact their ability to achieve their mission in the communities they serve.^1^ At the same time, rural social determinants of health are too complex for any single organization to address alone. Network-based approaches draw on the unique characteristics of rural communities by effectively leveraging the strengths of each partner and widening the net of resources which can foster self-organization, collective action, and sustainable change.^2^,^3^

Building on this framework, grassroots leaders in Hawkins and Hancock Counties, Tennessee, joined together in March 2021 to form a network of local leaders called ***Connections***. Connections was designed to create a space where faith-based and nonprofit leaders can grow meaningful relationships, coalesce around common interests, and engage in strategic thought partnerships to advance community-led action and place-based strategies for addressing health-related inequities. The work is guided by a six-member steering committee comprised of local nonprofit leaders.

Short-term goals of the network center on deepening the capacity of the network and member organizations and promoting partnerships as a vital condition for addressing rural social determinants of health. Longer term, network members will work together to influence local policies and priorities affecting residents.

To support these goals, Connections is providing network members with capacity-building tools and resources to achieve their missions and sustain impact. Data collected from network members are guiding these efforts. A key initiative has centered on mobilizing volunteers to support the work of agencies in the network. Volunteers are extremely hard to recruit in rural areas. Yet, they are the lifeblood of local agencies. Connections recently launched a new volunteer website. The website (http://www.yourvolunteerconnection.com) is a digital guide where visitors can learn about local volunteer opportunities and the services provided by organizations.

Network members are also aligning around common goals. For example, an important aspect of the network is to address gaps in the social service safety net for older persons living in the two counties. Toward this end, network members are developing an electronic and print Aging Resources Guide for older adults, family members, and professionals to access information on local home and community-based services and supports. Network members have also co-authored two grant proposals aimed at mobilizing volunteers to address the nonmedical needs of older adults aging in place. The interactive, collaborative grant-writing process has helped strengthen relationships between network members.

In February 2022, Connections launched the Community Collaboration Mini-Grants Program (www.yourvolunteerconnection.com/minigrant) as a mechanism to advance multi-sectoral partnerships. The mini-grants program provides up to $2,000 in financial support for projects conducted by local nonprofit, faith-based and service organizations. Applicants were required to address one or more funding priorities (i.e., social support, food security, quality of life, and older adults or adults with disabilities) and must partner with a local organization to carry out the project. The network awarded a total of $23,000 to 12 nonprofit and faith-based organizations in its first year. The funds will be used for a wide range of projects, including basic needs assistance, farm-to-table initiatives, meals-on-wheels programs, home repairs, and clean water projects. The steering committee will use data collected from grantees to evaluate the impact of the projects in the community and overall network engagement and health. Results from this evaluation will help inform ongoing efforts to engage network members, leverage existing resources, and identify synergies that can accelerate improvements in community health and well-being.

More recently, Connections launched the Youth Engaged in Service (YES) Challenge (www.yourvolunteerconnection.com/yeschallenge). Through this challenge, Connections will award $500 grants to youth who want to make a difference in the lives of older adults. The YES Challenge is designed to amplify and support youth changemakers. Projects must be youth-driven and youth-designed.
